# The Application of Functional Magnetic Resonance Imaging in Type 2 Diabetes Rats With Contrast-Induced Acute Kidney Injury and the Associated Innate Immune Response

**DOI:** 10.3389/fphys.2021.669581

**Published:** 2021-06-29

**Authors:** Yanfei Li, Dafa Shi, Haoran Zhang, Xiang Yao, Siyuan Wang, Rui Wang, Ke Ren

**Affiliations:** ^1^Department of Radiology, Xiang’an Hospital of Xiamen University, Xiamen, China; ^2^Department of Basic Medical Sciences, School of Medicine, Xiamen University, Xiamen, China

**Keywords:** CI-AKI, DM, fMRI, immunity, NLRP3 inflammasome

## Abstract

**Aims:**

Contrast-induced acute kidney injury (CI-AKI) is the third most common in-hospital acquired AKI, and its mechanism is not fully clear. Its morbidity increases among populations with chronic kidney disease (CKD), older age, diabetes mellitus (DM), and so on. Immediate and effective noninvasive diagnostic methods are lacking, so CI-AKI often prolongs hospital stays and increases extra medical costs. This study aims to explore the possibility of diagnosing CI-AKI with functional magnetic resonance imaging (fMRI) based on type 2 DM rats. Moreover, we attempt to reveal the immune response in CI-AKI and to clarify why DM is a predisposing factor for CI-AKI.

**Methods:**

A type 2 DM rat model was established by feeding a high-fat and high-sugar diet combined with streptozotocin (STZ) injection. Iodixanol-320 was the contrast medium (CM) administered to rats. Images were obtained with a SIEMENS Skyra 3.0-T magnetic resonance imager. Renal histopathology was evaluated using H&E staining and immunohistochemistry (IHC). The innate immune response was revealed through western blotting and flow cytometry.

**Results:**

Blood oxygenation level-dependent (BOLD) imaging and intravoxel incoherent motion (IVIM) imaging can be used to predict and diagnose CI-AKI effectively. The *R*^2^*^∗^* value (r > 0.6, *P* < 0.0001) and *D* value (| r| > 0.5, *P* < 0.0001) are strongly correlated with histopathological scores. The NOD-like receptor pyrin 3 (NLRP3) inflammasome participates in CI-AKI and exacerbates CI-AKI in DM rats. Moreover, the percentages of neutrophils and M1 macrophages increase dramatically in rat kidneys after CM injection (neutrophils range from 56.3 to 56.6% and M1 macrophages from 48 to 54.1% in normal rats, whereas neutrophils range from 85.5 to 92.4% and M1 macrophages from 82.1 to 89.8% in DM rats).

**Conclusions/interpretation:**

BOLD and IVIM-*D* can be effective noninvasive tools in predicting CI-AKI. The innate immune response is activated during the progression of CI-AKI and DM will exacerbate this progression.

## Introduction

Post-contrast acute kidney injury (PC-AKI) refers to a decrease in renal function after intravascular administration of contrast medium (CM; iodinate based). The term was coined by the Contrast Media Safety Committee (CMSC) of the European Society of Urogenital Radiology (ESUR) to replace the previous term contrast-induced AKI (CI-AKI), which is reserved only for cases in which a casual relation can be confirmed between CM injection and renal damage (van der Molen et al., 2018a). Although the risk of PC-AKI has been considered overestimated in a series of meta-analyses (Kooiman et al., 2012; Moos et al., 2013), the condition still affects patients who receive CM, especially those who have impaired renal function or kidney disease, resulting in increased morbidity and mortality and in prolonged hospital stays (Kooiman et al., 2015; Mitchell et al., 2015). In addition, no clinical tests can noninvasively assess PC-AKI at present. Functional magnetic resonance imaging (fMRI) is a current useful technique that can be used to test lesions; this method obtains functional information rather than the traditional structural images. To date, studies on the mechanism of CI-AKI have focused mainly on reactive oxygen species (ROS) generation as a result of kidney hypoxia, alterations in hemodynamics, CM toxicity, and other mechanisms. Little attention has been paid to the immune responses associated with CI-AKI. Moreover, diabetes mellitus (DM) is becoming a common disease threatening human health, and DM increases the risk of PC-AKI (Hoste et al., 2018; van der Molen et al., 2018b). DM is a common cause of chronic kidney disease (CKD) and contributes to AKI. We hypothesized that some synergic steps participate in CI-AKI in the context of DM. In this study, we first test fMRI as a potential technique for CI-AKI assessment. Then, we investigated the potential role of the innate immune response in CI-AKI. Finally, we explore the association between DM and CI-AKI. As our research concentrated on the casual relationship between CM administration and renal injury, we use “CI-AKI” rather than “PC-AKI” throughout this article.

## Materials and Methods

### Overall Experimental Designing

**FIGURE 1:**
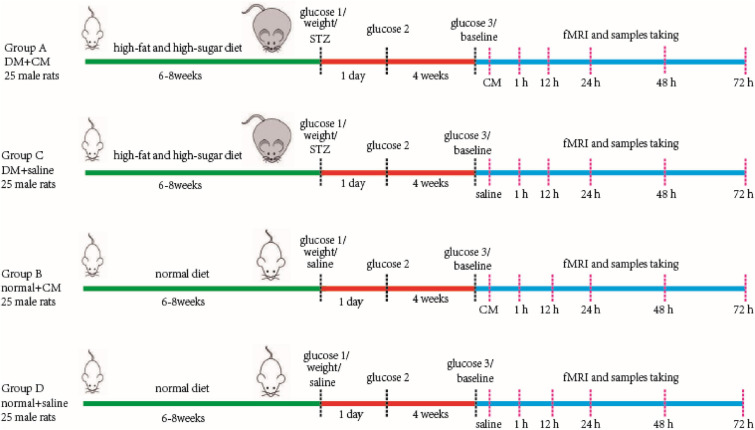
Main procedure of experiment workflow. Four groups of rats are performed as in the figure. Two random glucose test results at or above 16.7 mmol/L were the DM model criteria. Baseline scanning is prior to CM or saline tail injection. The serum and kidney samples were store at −80°C after collecting for further study. CM, contrast media; DM, diabetic mellitus; STZ, streptozocin.

One hundred male Sprague–Dawley (SD) rats aged 4–5 weeks were randomly divided into two primary groups. The rats in one primary group were used for the type 2 DM model, and the rats in the other were used as healthy normal controls. Then, each group was divided into two secondary groups composed of 25 rats each. Group A was the DM+CM group, Group B was the normal+CM group, Group C was the DM+saline group, and Group D was the normal+saline group. After a period of feeding and model establishment, all the rats underwent three main procedures, including tail injection of CM or saline, imaging, and sample collection. More specific information is given in the workflow schematic in Figure 1.

### Animal Model of Type 2 Diabetes Mellitus

Male SD rats aged 4–5 weeks were raised under suitable conditions at the Xiamen University Laboratory Animal Center under 12-h/12-h day/night cycles, a temperature of 22 ± 2°C, and a relative humidity of 50–70%. High-fat and high-sugar feed was provided to Group A and Group C for 6–8 weeks to induce obesity prior to type 2 DM. The feed composition is shown in Supplementary Table 1. The high-fat diet-fed rats (Group A and Group C) were much heavier than the regular diet-fed rats. The body weight changes are shown in Supplementary Figure 1.

The rats were fasted for 24 h, and their glucose levels were tested before intraperitoneal injection of streptozotocin (STZ). STZ buffer was prepared. Briefly, solution A was created by dissolving 2.1 g of citric acid in 100 ml of double-distilled water, and solution B was created by dissolving 2.94 g of trisodium citrate dihydrate in 100 ml of double-distilled water. Solutions A and B were mixed in equal volume, and the pH was adjusted to 4.2–4.5. Then, the solution was purified with a 0.45-μm filter to remove impurities and to obtain the STZ buffer. STZ was intraperitoneally injected (35 mg/kg; Dhuria et al., 2015) immediately after 1% (w/v) STZ was prepared. All these procedures were performed at 0–4°C without light. Blood was taken from the tail for glucose testing at least 24 h after STZ injection. Two random glucose test results at or above 16.7 mmol/L were the DM model criteria. The rats with high glucose status (HGS) were maintained for at least 4 weeks before subsequent experimentation. The glucose changes are shown in Supplementary Figure 2.

### Kidney Functional Magnetic Resonance Imaging

**FIGURE 2:**
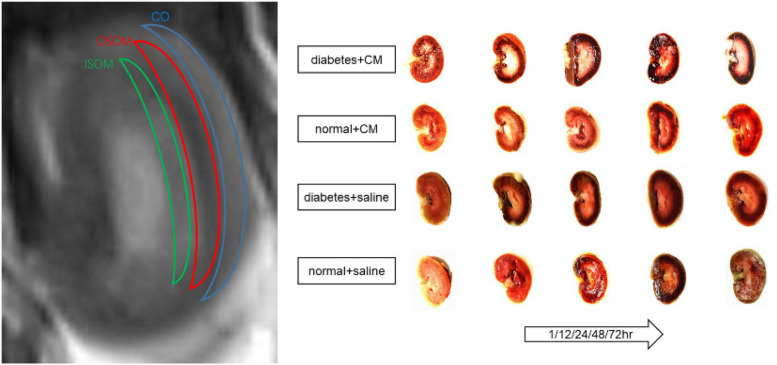
ROI on T2WI and kidney coronal section. From outside to inside are cortex (CO), outer stripe of the outer medulla (OSOM), and inner stripe of the outer medulla (ISOM). Coronal section of kidney of each group at different times is shown. CM, contrast medium; T2WI, T2 weighted image; ROI, region of interest.

Images were obtained with a SIEMENS Skyra 3.0-T magnetic resonance imager with a transmit coil array and a TxRX_Knee_15 coil. The parameters and sequences are in Table 1 (Chen et al., 2015; Liang et al., 2016; Wang et al., 2017, 2018; Zhang et al., 2018). Iodixanol-320 (4 gI/kg) (Lenhard et al., 2013; Wang et al., 2014) or an equal amount of saline was administered through the tail vein. At 1, 12, 24, 48, and 72 h after administration, rats were anesthetized with pentobarbital sodium (3%, w/v), and the coronal renal region was scanned in the prone position with the head in first. The SIEMENS workstation system was manipulated to view blood oxygenation level-dependent (BOLD) images and obtain manual regions of interest (ROIs), and MITK Diffusion software^1^ was used to view intravoxel incoherent motion (IVIM) images and obtain manual ROIs. All ROIs and the related quantitative regional IVIM parameter (*D*, *D*^∗^, and *f*) and BOLD parameter (*T*2*^∗^*) measurements were performed by two experienced radiologists who were blinded to the experimental conditions. The *D* value represents the slow diffusion coefficient (the pure molecular diffusion), the *D*^∗^ value represents fast diffusion coefficient (the flow velocity), and the *f* value represents the perfusion fraction (the fraction of water flowing in capillaries) (Mao et al., 2018; Hu et al., 2019). The BOLD parameters indicate the partial oxygen pressure determined by estimating the deoxyhemoglobin concentration in the kidneys (Prasad et al., 1996). The final BOLD parameters *R*2*^∗^* equals 1/*T*2*^∗^* numerically. The IVIM images were arranged to fit *D* and *f* (high b) and then to fit *D*^∗^. The ROIs on T2-weighted imaging (T2WI) images and representative gross renal specimens are shown in Figure 2.

**TABLE 1:** Parameters and sequences of IVIM and BOLD.

**Parameters/sequences**	**IVIM**	**BOLD**
Number of slices, n	3	4
Section thickness, mm	2.4	2
Repetition time, ms	3000	92
Echo time, ms	66	3.6
Orientation	Coronal	Coronal
Bandwidth, hertz perpixel	1,185	320
Field of view, mm2	86 × 114	60 × 120
Matrix	90 × 72 (144 × 192)	256 × 128
Number of excitations, n	1	10
Acquisition time, s	193.149	194.218
b-values (s/mm^2^)	0,20,40,60,80,100,200,400, 500,600	–
Flip angle	90	30
Breathing protocol	Free	Free

*IVIM, intravoxel incoherent motion; BOLD, blood oxygenation level-dependent.*

### Sample Collection

Soon after fMRI images were obtained, the rats were sacrificed with an overdose of anesthetics for 10 min. Blood from the eye socked and abdomen aorta was collected into tubes containing separation gel and coagulant and then was centrifuged at 4°C and 4,000 rpm for 10 min. The serum was collected into new cryogenic vials and stored at −80°C. The kidneys were removed by excision at the renal hilus, and their weights are shown in Supplementary Figure 3. The left kidney was stored for protein extraction at −80°C, and the right kidney was fixed for paraffin sectioning in 4% paraformaldehyde for 48 h.

### Hematoxylin and Eosin Staining

After the kidneys were fixed in 4% paraformaldehyde, they were dehydrated with an increasing gradient of ethanol and dimethylbenzene. Paraffin sections were cut at 4-μm thickness. H&E staining was performed with a kit according to the manufacturer’s instructions (H&E Staining Kit, C0105S, Beyotime, China). The specimens were scored according to the presence of tubular desquamation, necrosis, atrophy, cytoplasmic vacuoles, and interstitial infiltration. The renal lesions were graded under a microscope, as follows: 0, normal kidney tissue; 1, minimal injury (0–5%); 2, moderate injury (5–25%); 3, intermediate injury (25–75%), and 4, severe injury (75–100%) (Colbay et al., 2010).

### Immunohistochemistry

After deparaffinization, antigen retrieval was performed in 100°C citrate antigen retrieval solution for 15 min, and the samples were cooled to room temperature gradually. H_2_O_2_ (3%) was used to block the endogenous peroxidase activity. The sections were then incubated with a polyclonal anti-hypoxia-inducible factor (HIF)-1α primary antibody (1:100, ab1, Abcam, United Kingdom) at 4°C overnight and with a horseradish peroxidase (HRP)-labeled secondary antibody for 30 min. Diaminobenzidine (DAB) was used to reveal the expression of HIF-1α. The intensity of HIF-1α staining was evaluated by the H-score assessment. The H-score, which was calculated by adding the percentages of positive cells with four different staining intensities (SIs) multiplied by the different Sis, ranged from 0 to 300 points. A sample with an H-score ≥50 points was considered positive. H-score = [1 × (% cells 1+) + 2 × (% cells 2+) + 3 × (% cells 3+)] (Specht et al., 2015). Two independent pathologists who were blinded to the experimental conditions completed the assignment according to the above criteria.

### Serum Enzyme-Linked Immunosorbent Assay

The amounts of blood urea nitrogen (BUN), serum creatinine (Scr), interleukin-18 (IL-18), and interleukin-1β (IL-1β) were tested with serum enzyme ELISA at Wuhan Servicebio Technology Co., Ltd.

### Protein Extraction and Western Blotting

One kidney from each rat was placed in a proportionate amount (mg/μl/μl, 20:100:1) of ice-cold lysis buffer with phenylmethylsulfonyl fluoride. The kidney was cut into pieces, and the debris was homogenized with ultrasonication. The sample was centrifuged at 4°C and 12,000 rpm for 10 min, and then the supernatant was collected. A proportionate amount of sodium dodecyl sulfate (SDS) loading buffer was added to the extract, and the mixture was blended. The protein was denatured at 95°C for 10 min. The sample was stored at −80°C or used to test the protein concentration with bicinchoninic acid (BCA) method so that equal concentrations of protein could be loaded for Western blotting. Approximately 10 μl of each sample was added to an 8 or 12% SDS–polyacrylamide gel, and the samples were electrophoresed at 120 V for 50 min. Thereafter, the proteins were transferred onto a nitrocellulose filter membrane at 90 V for 100 min. Nonspecific binding sites were blocked with 5% skim milk powder at room temperature for 60 min, and the membrane was then incubated with primary antibodies against NOD-like receptor pyrin 3 (Nlrp3) (1:1,000, NBPZ-12446, Novus Biologicals, United States), caspase-1 (1:1,000, ab179515A, Abcam, United Kingdom), caspase-8 (1:1,000, #4790, Cell Signaling Technology, United States), and GAPDH (1:1,000, 10494-1-AP, Proteintech, China) at 4°C overnight. After being washed with TBST buffer, the membrane was incubated with HRP-conjugated secondary antibodies at room temperature for 60 min. The immunostained protein bands were visualized with a chemiluminescence system. GAPDH was the internal control.

### Flow Cytometry

Rat kidneys were removed from renal hilum after anesthetization. Half of the corona of each kidney was removed, cut into pieces, and collected into tubes. Type IV collagenase (abs47048003, Absin Bioscience, 100 mg/ml) and deoxyribonuclease (D4527-10KU, Sigma, 50,000 kU/ml) were added to these tubes, and the tubes were incubated for 30 min at 37°C. The pellets were filtered with a 70-μm strainer and then centrifuged at 4°C for 10 min. The pellet was collected, and ammonium–chloride–potassium (ACK) lysis buffer was added for erythrolysis for 15 min at 4°C. The sample was again centrifuged for 10 min at 4°C, and the pellet was resuspended with 40% Percoll. The suspension was poured slowly down the inner wall of a 15 ml tube with 80% Percoll so that the different concentrations of Percoll clearly separated. A third centrifugation step was performed for 20 min. The middle layer between 40% and 80% Percoll contained target leukocytes. The middle layer was collected and centrifuged for 5 min. The pellet was then collected and suspended in 1 ml of PBS [2% fetal bovine serum (FBS), v/v]. The cells were blocked with an anti-FcγII antibody (CD32, 1:200, 550270, BD Pharmingen, United States) at 4°C for 15 min. The Fc antibody was rinsed off. The cells were then incubated with CD marker antibodies (CD45, 1:200, 565465, BD Pharmingen, United States; CD11b/c, 1:200, 562222, BD Pharmingen, United States; CD18, 1:200, 744907, BD Pharmingen, United States; CD86, 12-0860-83, eBioscience, United States) for 20 min at 4°C in darkness and analyzed by flow cytometry.

### Statistical Analysis

All the values were calculated and analyzed with SPSS 20.0 or GraphPad Prism 5. The outcomes are expressed as the mean ± standard deviation. If the data were normally distributed (body weight, kidney weight, and glucose), one-way analysis of variance (ANOVA) or repeated-measures ANOVA was performed to test the differences, and Bonferroni post hoc tests were performed for further comparison. If the data were not normally distribution (fMRI values, histological scores, H-scores, BUN, Scr, and cytokine quantities), the Kruskal–Wallis test, Friedman test, and Mann–Whitney U test were performed for intergroup or intragroup comparisons. Spearman correlation analysis was used to study the linkage between fMRI and pathology.

## Results

### Functional Magnetic Resonance Imaging

#### Blood Oxygenation Level-Dependent

**FIGURE 3:**
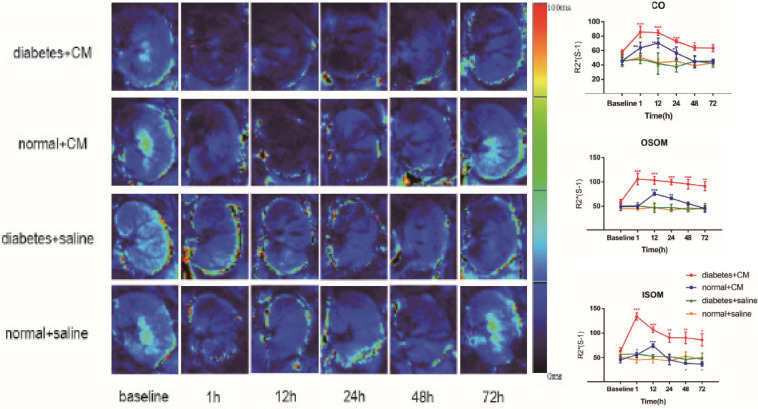
Parametric images of BOLD images. Four groups of images from up to down are the representative T2* images. Group A Diabetes+CM, Group B Normal+CM, Group C Diabetes+saline, Group D Normal+saline. The parameter stands for T2* in these images which are transferred to its reciprocal *R*2* in the following three line charts. (Compared with baseline, *P < 0.05, **P < 0.005, ***P < 0.0001). CO, Cortex; OSOM, outer stripe of the outer medulla; ISOM, inner stripe of the outer medulla; CM, contrast medium; BOLD, blood oxygenation level-dependent.

The BOLD images and *R*2*^∗^* graphs are presented in Figure 3 and all the *R*2*^∗^* values are in Table 2. In Groups A and B, *R*2*^∗^* increased in the first 1 h after administration of iodixanol, and the color became darker at 24 h in Groups A and B than in Groups C and D. Group B recovered faster and more comprehensively than Group A within 72 h. Although Groups C and D were injected with saline, the parametric images showed slight changes; however, the changes were not as obvious as those in Groups A and B. Wherever an ROI was located in these graphs, after administration of iodixanol, *R*2*^∗^* (red and blue line) presented an up-and-down tendency. Notably, the value for Group A peaked at 1 h, and the value for Group B peaked at 12 h. Together with the BOLD images, which show the underlying recovery time, these findings might imply that DM or HGS is a predisposing factor for hypoxic injury. The *R*2*^∗^* difference between Groups A and B after administration of iodixanol was obvious (*P* < 0.005) in all three ROIs, indicating that HGS is a potential inducer to CI-AKI. In contrast, the lack of obvious difference between Groups C and D (*P* > 0.1) indicates that DM or HGS alone cannot induce CI-AKI in animal models; CM is also needed to activate the renal damage progression.

**TABLE 2:** Values of parametric image from four groups of CO, OSOM, and ISOM.

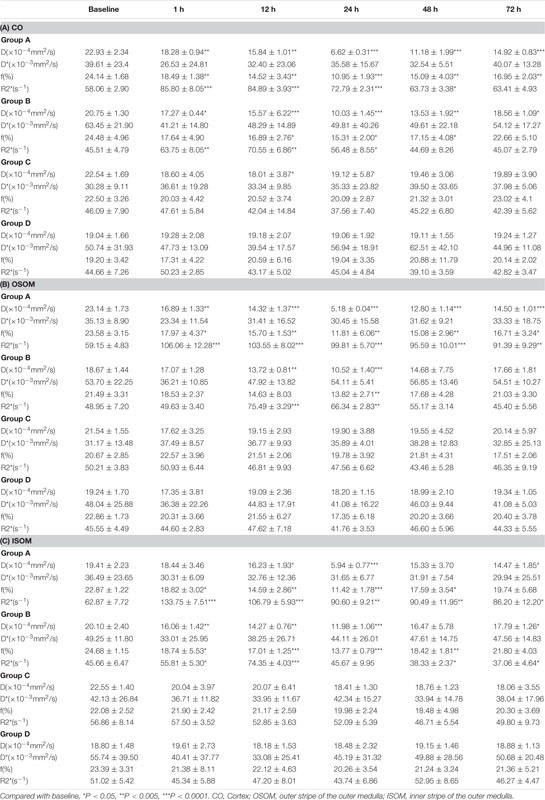

Compared with baseline, *P < 0.05, **P < 0.005, ***P < 0.0001. CO, Cortex; OSOM, outer stripe of the outer medulla; ISOM, inner stripe of the outer medulla.

#### Intravoxel Incoherent Motion

**FIGURE 4:**
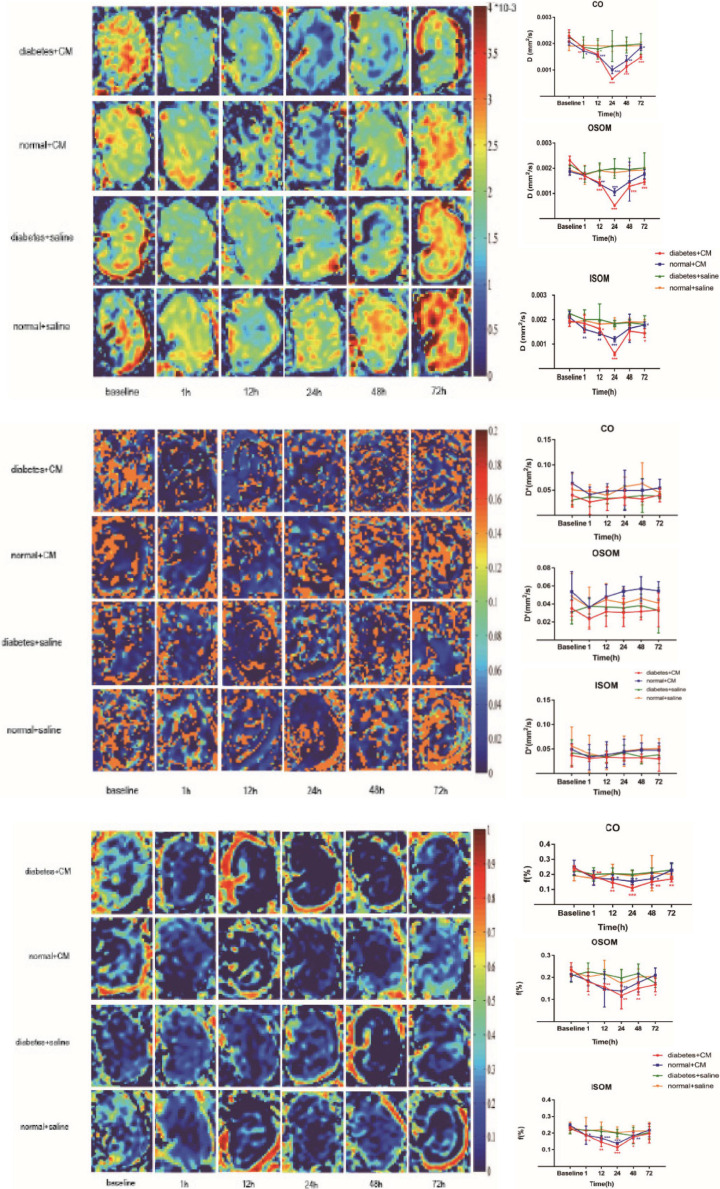
Parametric images of IVIM images. The three clusters of images are the representative *D*, D*, and *f* images. Group A diabetes+CM, Group B normal+CM, Group C diabetes+saline, and Group D normal+saline. Compared with baseline, *P < 0.05, **P < 0.005, ***P < 0.0001. CO, cortex; OSOM, outer stripe of the outer medulla; ISOM, inner stripe of the outer medulla; CM, contrast medium; IVIM, intravoxel incoherent motion.

The IVIM images and *D*, *f*, and *D*^∗^ graphs are shown in Figure 4 and all the *D, f*, and *D*^∗^ values are in Table 2. The *D* value represents pure water diffusion. After tail injection of CM, the *D* value began declining after the first hour and reached its lowest value at 24 h. Then, the value gradually increased to close to the normal level. The value for Group A had a sharper decline than that for Group B in the second 12 h and subsequently recovered more slowly than the value for Group B. The distinction between Groups A and B became strong at 24 h (*P* < 0.005) and gradually returned to the previous level, but no difference between Groups C and D was detected (*P* > 0.1). The *D*^∗^ value, which represents flow velocity, seemed less effective than *D* value as an indicator of renal injury because the *D*^∗^ value drifted above and below the baseline in the three graphs and because no differences were observed (*P* > 0.1). In addition, Groups C and D had a clearly lower *D*^∗^ than Group B in the outer stripe of the outer medulla (OSOM), which probably means that CM has little effect on flow velocity in the kidneys. In addition, the overall outline of the kidneys in the *D*^∗^ images was not as clear as that in the *D* or *f* images. The *f* value, which represents the fraction of water flowing in capillaries, appeared as effective as *D* for evaluation of kidney damage caused by CM. Groups A and B showed almost the same alterations after CM injection; the minimum value was observed at 24 h, and the difference was the greatest between the groups at 24 h (*P* < 0.01). Thereafter, the values tended to return to baseline. In summary, according to the IVIM images, *D* is the most useful value for evaluation of renal injury, especially for judgment of whether DM can increase the incidence of CI-AKI. According to the *D* and *f* graphs at 24 h, the ROI most vulnerable to CM may be the OSOM, followed by the cortex (CO) (*P* < 0.01).

### Histopathology

**FIGURE 5:**
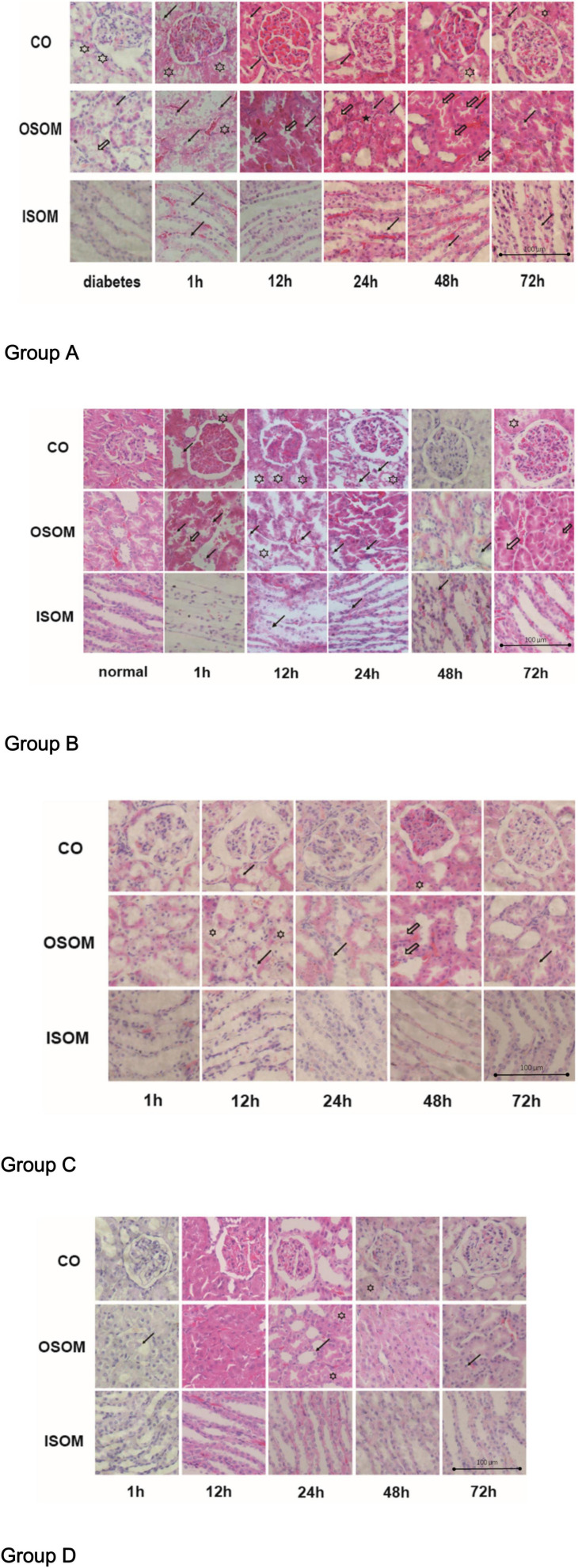
Histological pathology of kidney, ×400. Group A diabetes+CM, Group B normal+CM, Group C diabetes+saline, and Group D normal+saline. The hollow stars show cytoplasmic vacuoles. The hollow arrows present the proteinaceous cast. The black stars show interstitial infiltration. The black arrows indicate tubular desquamation, necrosis, or atrophy. CO, cortex; OSOM, outer stripe of the outer medulla; ISOM, inner stripe of the outer medulla; CM, contrast medium.

The pathological changes in the kidneys are presented in H&E-stained sections in Figure 5.

For Group A, the H&E images from left to right show three areas of the kidneys—the CO, the OSOM, and the inner stripe of the outer medulla (ISOM)—at 1, 12, 24, 48, and 72 h after administration of CM. In the first 1 h after CM injection, cytoplasmic vacuoles appeared in tubular cells, and nuclei began to fragment. By 12 h, the spaces between tubular cells had widened, small proteinaceous casts had appeared, and the nuclei had fragmented more obviously. By 24 and 48 h, the proteinaceous casts had become larger, and interstitial infiltration was observed outside the tubules, which made the lumens smaller and narrower. By 72 h, some of the tubules remained active, the intracellular spaces had decreased in size, and the proteinaceous casts had begun to disappear. Notably, the glomeruli were much less affected than tubules histologically in this group. Compared with Group A, Group B exhibited fewer cytoplasmic vacuoles in the tubular cells in the first 1 h. Twelve hours after CM injection, cytoplasmic vacuoles had begun appearing in tubular cells, but the tubules and nuclei had fragmented earlier, by 1 h. In addition, proteinaceous cast formation was much lower in Group B than in Group A and was alleviated sooner in Group B than in Group A at 48 h; this finding may indicate that DM is an activator of CI-AKI. The renal injury in Group C was obviously milder than that in Group A. In the first 1 h after saline injection, almost no injury was apparent in the three regions of the kidneys in Group C. At 12 h, tubular epithelial desquamation and cytoplasmic vacuolation were sparsely observed. After 24 h, the damaged tubules had recovered, and there were much lower amounts of proteinaceous casts in Group C than in Group A. At 72 h, the renal morphological features were nearly the same as those at 1 h. Pathological changes were less noticeable in Group D than in Group B, but tubular edema and lumen narrowing appeared at 12 h. Almost no proteinaceous casts were found in tubules. At 24 h, a small portion of the tubules exhibited desquamated epithelia, and little interstitial infiltration was observed. It was unclear if these changes were due to saline injection. In Group A and Group B, the OSOM exhibited the most severe pathological changes among the ROIs (Hu et al., 2019).

**FIGURE 6:**
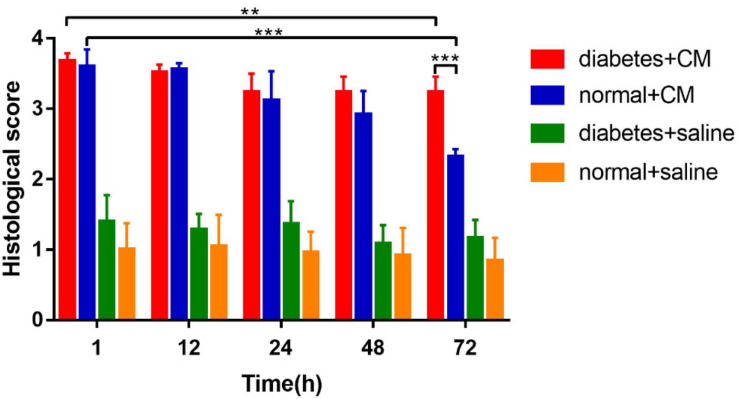
Histological score. *P < 0.05, **P < 0.005, ***P < 0.0001.

The calculated histological scores are shown in Figure 6.

Group A had the most critical damage of all groups even until 72 h. Although Group B exhibited injury as severe as that of Group A on the first day, it recovered by 72 h; and the histological score dropped dramatically in 72 h in Group B compared with Group A. The difference between 1 and 72 h in Group A was smaller than that in Group B, which reveals that HGS may prolong the recovery time for CI-AKI or even lead to irreversible kidney damage when combined with CM-induced injury. Groups C and D had very similar scores from the beginning, which indicated that no significant pathological changes occurred under saline conditions, although the score for Group C was consistently slightly higher than that Group D. In summary, CM seemed to affect renal injury more strongly than HGS in our animal models, indicating that HGS increases susceptibility to CI-AKI and impedes recovery.

### Hypoxia-Inducible Factor-1α

**FIGURE 7:**
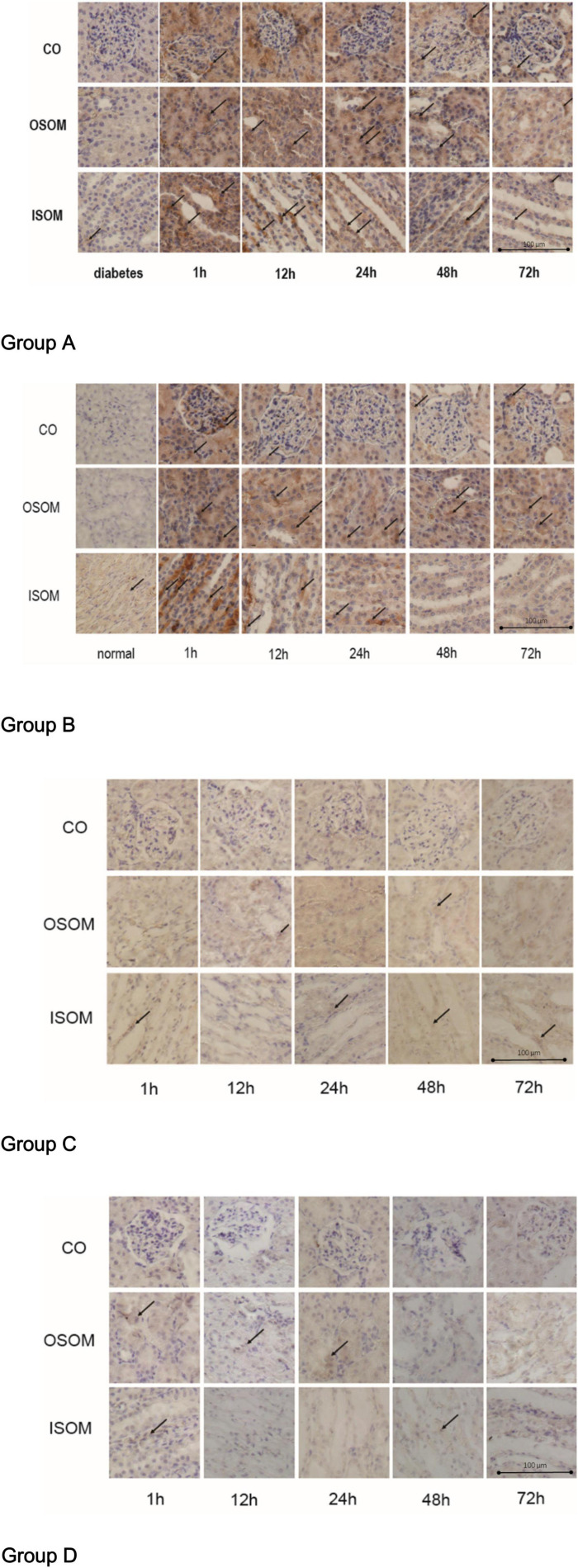
HIF-1α expression in kidney, ×400. Group A diabetes+CM, Group B normal+CM, Group C diabetes+saline, and Group D normal+saline. The arrows show the strongest staining in one representative scope. CO, cortex; OSOM, outer stripe of the outer medulla; ISOM, inner stripe of the outer medulla; CM, contrast medium; HIF-1α, hypoxia-inducible factor-1α.

The results for HIF-1α expression in the kidneys are presented in Figure 7.

The immunohistochemistry (IHC) images for Group A, from left to right, show HIF-1α expression in three areas—the CO, the OSOM, and the ISOM—at 1, 12, 24, 48, and 72 h after administration of CM. The strongest staining appeared in the first 1 h, and staining did not totally decrease until 72 h. In Group B, the tendency of SI was the same as Group A. The darkest staining appeared in the first 1 h, but the staining seemed to decrease earlier in Group B than that in Group A. In Group C, the SI was weaker than that in Group A and Group B, indicating that CM, rather than HGS, increased HIF-1α expression. In Group D, almost no HIF-1α was expressed.

**FIGURE 8:**
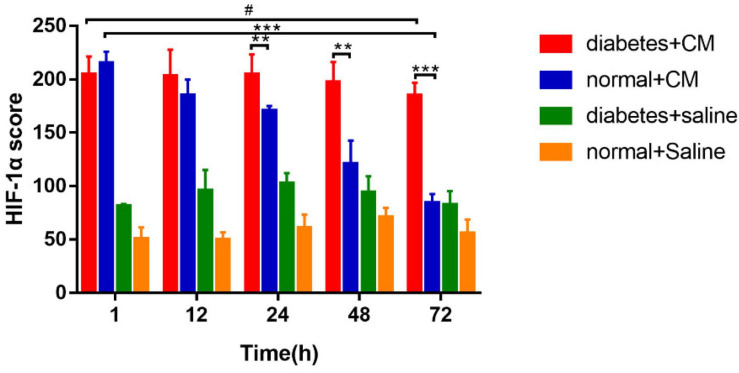
HIF-1α score. *P < 0.05, **P < 0.005, ***P < 0.0001, ^#^*P* > 0.05. HIF-1α, hypoxia-inducible factor-1α.

The calculated H-scores for HIF-1α are shown in Figure 8.

Group A had the highest score from 1 to 72 h, and the difference between the two time points was not significant. Group B had a score similar to that of Group A at the 1 h, but the score declined faster in Group B than in Group A after 12 h. Similar to the case for the histological scores, the difference between 1 and 72 h was much less significant for Group A than for Group B. These data indicate that HGS may have a stronger effect on HIF-1α expression than on the histological score according to the column heights of Group C and Group D in Figures 6, 8.

### Correlation Between Functional Magnetic Resonance Imaging and Pathology

#### *R*2*^∗^* vs Histology and Hypoxia-Inducible Factor-1α

**FIGURE 9:**
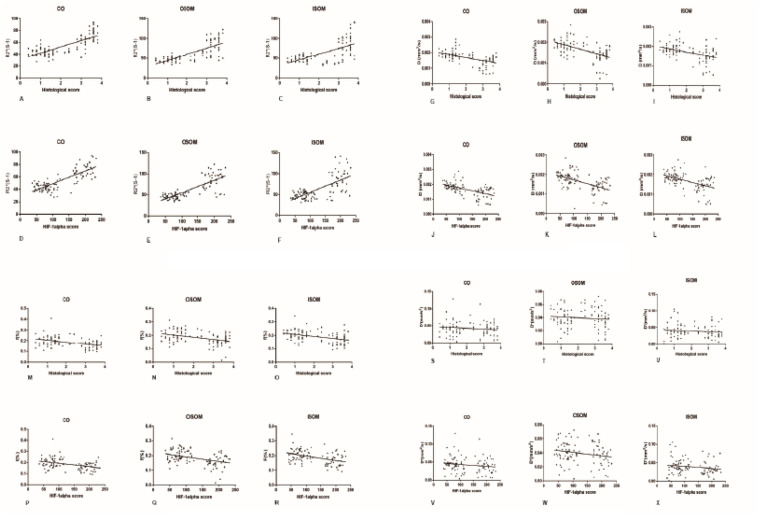
Correlation between fMRI and pathology. **(A-F)** *R*2* vs histology and HIF-1α: **(A)** r = 0.7605, **(B)** r = 0.7546, **(C)** r = 0.6331, **(D)** r = 0.7926, **(E)** r = 0.7824, and **(F)** r = 0.6952 (*P* < 0.0001). **(G-L)** *D* vs histology and HIF-1α: **(G)** r = −0.515, **(H)** r = −0.5356, **(I)** r = −0.4498, **(J)** r = −0.559, **(K)** r = −0.5261, and **(L)** r = −0.5123 (*P* < 0.0001). **(M-R)** *f* vs histology and HIF-1α: **(M)** r = −0.3858, **(N)** r = −0.4021, **(O)** r = −0.3981, **(P)** r = −0.3828, **(Q)** r = −0.4144, and **(R)** r = −0.4357 (*P* < 0.0001). **(S-X)** D* vs histology and HIF-1α: **(S)** r = 0.1171, **(T)** r = −0.09212, **(U)** r = −0.1064, **(V)** r = −0.162, **(W)** r = −0.1996, and **(X)** r = −0.1764 (*P* > 0.05). HIF-1α, hypoxia-inducible factor-1α.

A strong positive correlation was found between *R*2*^∗^* and the histological score (CO, r = 0.7605, *P* < 0.0001; OSOM, r = 0.7546, *P* < 0.0001; ISOM, r = 0.6331, *P* < 0.0001). The correlation between *R*2*^∗^* and HIF-α expression was as strong as that between *R*2*^∗^* and the histological score (CO, r = 0.7926, *P* < 0.0001; OSOM, r = 0.7824, *P* < 0.0001; ISOM, r = 0.6952, *P* < 0.0001; see Figures 9A-F). More severe kidney damage is associated with higher *R*2*^∗^* values. These results imply that renal hypoxia is a main inducer of renal injury in CI-AKI.

#### *D* vs Histology and Hypoxia-Inducible Factor-1α

A moderate negative correlation was calculated between the *D* value and histological score (CO, r = −0.515, *P* < 0.0001; OSOM, r = −0.5356, *P* < 0.0001; ISOM, r = −0.4498, *P* < 0.0001). The correlation between the *D* value and HIF-α expression was also moderate (CO, r = −0.559, *P* < 0.0001; OSOM, r = −0.5261, *P* < 0.0001; ISOM, r = −0.5123, *P* < 0.0001). These results are shown in Figures 9G-L.

#### *f* vs Histology and Hypoxia-Inducible Factor-1α

A weak negative correlation was calculated between the *f* value and histological score (CO, r = −0.3858, *P* < 0.0001; OSOM, r = −0.4021, *P* < 0.0001; ISOM, r = −0.3981, *P* < 0.0001). The correlation between the *D* value and HIF-α was also moderate (CO, r = −0.3828, *P* < 0.0001; OSOM, r = −0.4144, *P* < 0.0001; ISOM, r = −0.4357, *P* < 0.0001). These results are shown in Figures 9M-R.

#### *D*^∗^ vs Histology and Hypoxia-Inducible Factor-1α

No correlation was calculated between the *D*^∗^ value and histological score (CO, r = −0.1171, *P* = 0.2485; OSOM, r = −0.09212, *P* = 0.3620; ISOM, r = −0.1064, *P* = 0.2919). There was also no correlation between the *D* value and HIF-α expression (CO, r = −0.162, *P* = 0.1073; OSOM, r = −0.1996, *P* = 0.0465; ISOM, r = −0.1764, *P* = 0.0792). These results are shown in Figures 9S-X.

### Blood Urea Nitrogen and Serum Creatinine

**FIGURE 10:**
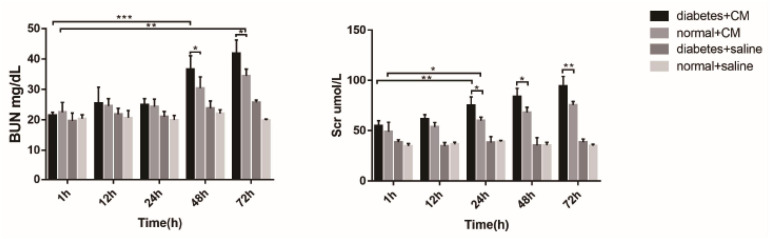
BUN and Scr. *P < 0.05, **P < 0.005, ***P < 0.0001. BUN, blood urea nitrogen; Scr, serum creatinine.

The BUN levels of both Group A and Group B increased gradually, in contrast to those of Group C and Group D, which revealed that CI-AKI developed to some extent (see Figure 10). An obvious difference in Group A was observed at 48 h (*P* < 0.0001), and an obvious difference in Group B was observed at 72 h (*P* < 0.005). On the first day after CM injection, no differences were found in Groups A and B. The differences appeared at 48 h (*P* < 0.05) and were still present at 72 h (*P* < 0.05), which might indicate that HGS is associated with CI-AKI-related damage capacity. No differences were found in Groups C and D, which likely indicated that HGS alone had little or no effect on renal injury in our experiment.

The increase in Scr was similar to the alterations in BUN, but the differences became apparent earlier for Scr than for BUN. At 24 h, the difference in Group A (*P* < 0.005) was greater than in Group B (*P* < 0.05). The difference between Groups A and B became clear at 24 h (*P* < 0.05) and expanded until 72 h (*P* < 0.005). Again, no differences existed in Groups C and D in this experiment. Overall, BUN and Scr exhibited almost the same value as CI-AKI indicators, but Scr may be more sensitive than BUN. The results of urine microalbumin of each group are shown as another indicator for renal injury in Supplementary Figure 4.

### Interleukin-1β and Interleukin-18

**FIGURE 11:**
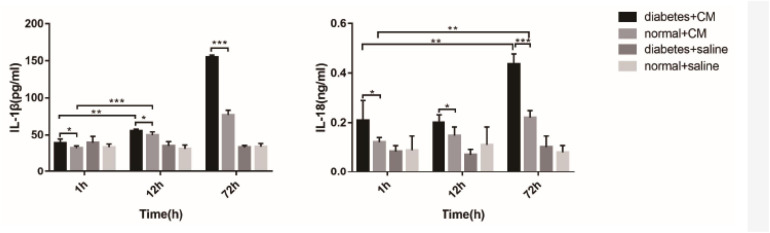
IL-1β and IL-18 in serum. *P < 0.05, **P < 0.005, ***P < 0.0001. IL-1β, interleukin-1β; IL-18, interleukin-1β.

Interleukin-1β and interleukin-18are the two cytokines related to Nlrp3-mediated pyroptosis. Figure 11 shows that the concentrations were different among the four groups. A difference in IL-1β level between Groups A and B was found at 1 h after CM administration (*P* = 0.0406), and this difference was enhanced at 72 h (*P* < 0.0001). At 12 h, the difference between Groups A and B was not as obvious (*P* < 0.05) as that at 72 h (*P* < 0.0001). IL-18 seemed less sensitive than IL-1β in our experiment because the differences in Group A and B occurred at 72 h (A, *P* = 0.0005; B, *P* = 0.0002). The difference in IL-18 between Groups A and B was similar to the difference in IL-1β; it began at 1 h (*P* < 0.05) and was enhanced at 72 h (*P* < 0.0001).

### Western Blotting

**FIGURE 12:**
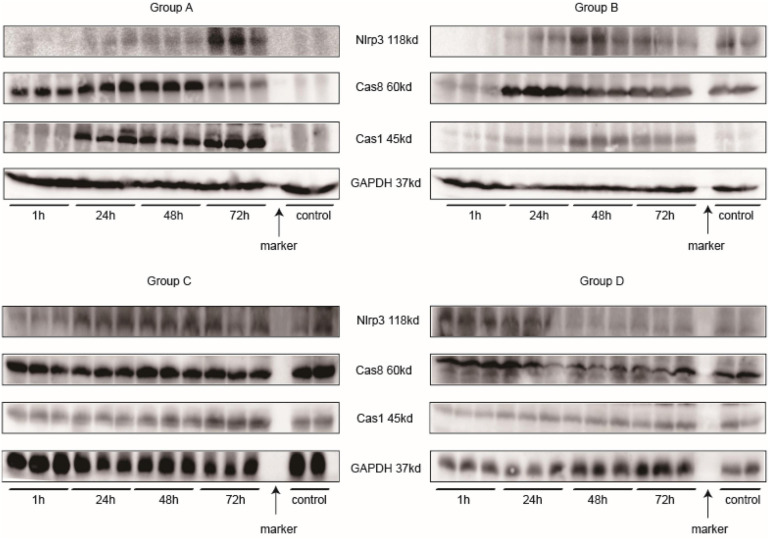
Western blotting. Every three samples next to each other on the left of the marker belong to the same time point. The two samples on the right of the marker are controls.

Main related protein in this study of each group are in Figure 12. Nlrp3 expression was most highly expressed at 72 h in Group A, and caspase-1 had the same tendency. In Group B, Nlrp3 was predominantly expressed at 48 h, and its expression was decreased at 72 h; the results for caspase-1 were consistent with those for Nlrp3. Although we did not determine how Nlrp3 was altered after 72 h in Group A, the band in Group A looked darker than that in Group B for the same sample. Caspase-8 was not expressed at the same time as caspase-1 or Nlrp3; it was expressed somewhat earlier. The strongest band for caspase-8 appeared 24 h before the strongest bands for Nlrp3 and caspase-1. Together with IL-18 and IL-1β levels, NLRP3 inflammasome levels seemed to peak at 48–72 h after injection of iodixanol-320. DM status could enhance this process and strengthen immune damage to the kidney.

### Flow Cytometry

**FIGURE 13:**
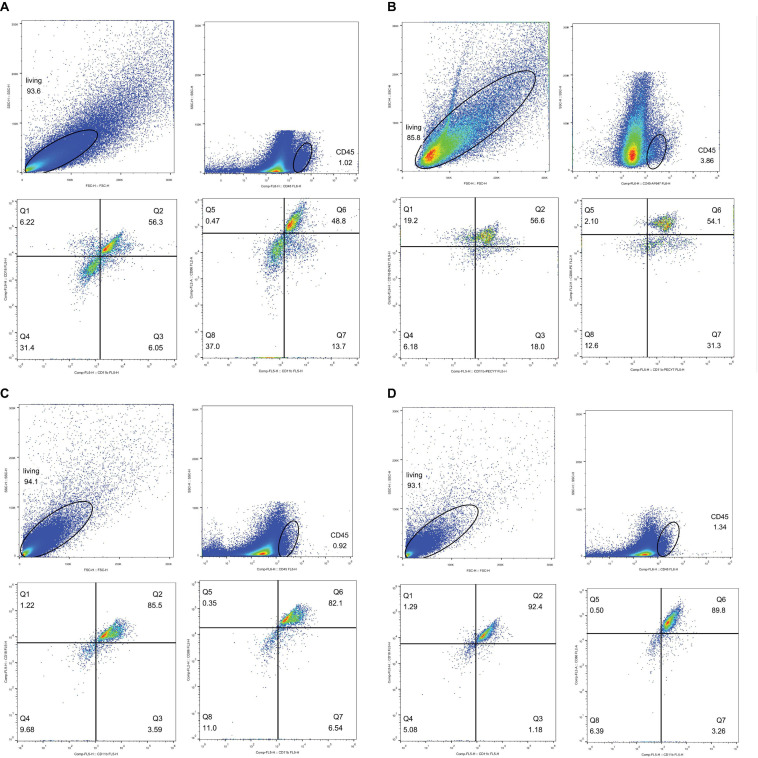
Flow cytometry analysis. Neutrophil, CD45^+^CD11b/c^+^CD18^+^; M1 macropahge, CD45^+^CD11b/c^+^CD86^+^. Gate setting: living cells→CD45^+^→CD11b/c^+^→CD18^+^ or CD86^+^. **(A)** Neutrophil, normal rat, Q2: CD45^+^CD11b/c^+^CD18^+^, accounting for 56.3%. M1 macrophage, normal rat, Q6: CD45^+^CD11b/c^+^CD86^+^, accounting for 48.8%. **(B)** Neutrophil, normal rat with contrast medium, Q2: CD45^+^CD11b/c^+^CD18^+^ accounting for 56.6%. M1 macrophage, normal rat with contrast medium, Q6: CD45^+^CD11b/c^+^CD86^+^ accounting for 54.1%. **(C)** Neutrophil, diabetes rats, Q2: CD45^+^CD11b/c^+^CD18^+^, accounting for 85.5%. M1 macrophage, diabetes rats, Q6: CD45^+^CD11b/c^+^CD86^+^, accounting for 82.1%. **(D)** Neutrophil, diabetes rats with contrast medium, Q2: CD45^+^CD11b/c^+^CD18^+^, accounting for 92.4%. M1 macrophage, diabetes rat with contrast medium, Q6: CD45^+^CD11b/c^+^CD86^+^, accounting for 89.8%.

It is essential to determine whether phagocytes participate in CI-AKI. According to Nlrp3 expression, samples from rats injected with CM were used for flow cytometry analysis 72 h after injection. CD45 is a leukocyte common antigen marker, and CD11b/c is a marker expressed on both neutrophils and macrophages. In addition, CD18 is a marker specific to neutrophils and CD86 is a marker for M1 macrophage (Tagzirt et al., 2014; Harwani et al., 2016; Rubio-Navarro et al., 2016). Figure 13A shows the normal rats without CM injection, and Figure 13B shows the normal rats with CM injection. The results in these figures demonstrate that the numbers of leukocytes were increased, the numbers of CD45^+^CD11b/c^+^CD18^+^ neutrophils were increased little at 72 h after CM injection, and the numbers of CD45^+^CD11b/c^+^CD86^+^ M1 macrophages were sharply increased. Figure 13C shows the results for DM rats without CM, and Figure 13D shows the results for DM rats with CM. Again, CM injection increased both neutrophil and M1 macrophage numbers, and the total numbers of these cells were much greater than the numbers of cells in the Q2 and Q6 gates, as shown in Figures 13A,B, respectively. We also made kidney IHC of CD3 and Ly6G in Supplementary Figure 5 to exclude the likelihood of T cell’s involvement.

## Discussion

The two primary intentions of our study were to monitor the progression of CI-AKI by fMRI and to reveal the possible innate immune response associated with this disease under DM conditions. We first established rats with DM and then administered CM to Groups A (DM+CM) and B (normal+CM) and saline to Groups C (DM+saline) and D (normal+saline) through tail vein injection. Groups C and D were essential for distinguishing whether DM or CM had a greater contribution to CI-AKI. The second stage involved fMRI scanning at different times and sacrificing of rats soon after that. Finally, animal samples were preserved and prepared for subsequent experiments. Samples from several additional rats were preserved for flow cytometry. Leukocytes, neutrophils, and macrophages were analyzed to study the extent to which the innate immune response is activated in CI-AKI.

The BOLD images in Figure 3 show the fMRI data used in our study to evaluate renal blood oxygen content and to predict CI-AKI. Upon comparing Groups A and C (or B and D), we concluded that the renal damage is induced by CM and that the main condition is hypoxia-related oxidative stress, which has been supported in various previous experiments (Caiazza et al., 2014; Wang et al., 2019; Kusirisin et al., 2020). Some clinical studies on CKD have obtained similar outcomes (Khatir et al., 2015; Hirakawa et al., 2017; Li et al., 2019). BOLD imaging is a strategy for evaluation of blood oxygenation, so *R*2*^∗^* can be used to predict CI-AKI (Wang et al., 2020). In addition, HGS may exacerbate oxygen consumption and renal damage to some extent given the prolonged recovery time in Group A, and the underlying mechanisms are likely multifactorial, involving far more than hypoxia (Heyman et al., 2013; Wada and Makino, 2016). Moreover, if hypoxia is irreversible, it results in continuous tubular or vascular impairment, which weakens normal renal function and affects water diffusion and perfusion, as discussed below. Our observation period ended at 72 h, so it is unclear if the *R*2*^∗^* values of Group A rats would have returned to normal at later time points. To demonstrate the reliability of BOLD imaging, we performed renal histopathology. We chose HIF-1α as a molecular marker for detection of oxygen levels because it is popular in studies on kidney disease, which is a closely related to hypoxia and renal anemia (Nangaku and Eckardt, 2007; Gunton, 2020; Voit and Sankaran, 2020). The expression of HIF-1α shown in Figure 7 led to similar conclusions. The correlations between *R*2*^∗^* values and HE scores or HIF-1α expression (Figures 9A-F) also validate this finding (discussed later).

IVIM imaging (Figure 4) is another fMRI method that reflects the movement of water molecules and the hydrodynamics of microcirculation. After administration of CM, both water diffusion and perfusion in the kidneys changed substantially. Although the *D* value is not as sensitive as the *R*2*^∗^* value in estimating renal injury in the context of CI-AKI, it can still be used as a predictor of CI-AKI due to its association with molecular diffusion. Of the three IVIM values in our study, *D* was the most effective, *f* was the second most effective, and *D*^∗^ was the least effective. The correlation graphs in Figures 9B-D clearly show that that *D* and *f* were more effective than *D*^∗^. CM alters water diffusion by changing metabolism through the kidneys and affects molecular diffusion in the kidneys; these changes are similar to those of CKD detected by IVIM (Mao et al., 2018). Diffusion (indicated by the *D* value) is the dominant alteration because it is the most specific and repeatable character (Hu et al., 2019). Here, we refer to several dynamic concepts that may be unfamiliar to medical practitioners. In the field of microscopy, substances can be transported through three mechanisms: diffusion, advection, and dispersion. Dispersion occurs after advection and is an advanced type of diffusion that takes place when solutes with various velocities coexist. Hydrodynamic dispersion = molecular diffusion + mechanical dispersion. When advection is minimal, diffusion dominates over dispersion; in contrast, when advection becomes stronger, dispersion dominates (Todd and Mays, 2003; Peter, 2005; Emami-Meybodi et al., 2015; Hussain, 2015). In studies on human body and in most medical references, molecular movement in the kidneys and other organs includes mainly two types: diffusion and perfusion. Physiologically, the dynamics of perfusion depend on blood flow in vessels, while the dynamics of diffusion depend much more on partial pressure gradients. The kidneys undergo swift hemodynamic alterations after tail vein injection of CM. We speculate that blood flow first slows due to the much higher viscosity and osmolality of CM than blood and due to the CM on cardiac preload and afterload. At the same time, CM starts to permeate in all directions and disturb normal ion movement. Therefore, in fMRI, the diffusion (*D* value) ≈ dispersion relationship indicates pure diffusion based on molecular diffusion from a high to low concentration, while perfusion (*D*^∗^ value) = advection relationship indicates intravoxel capillary perfusion-based molecular diffusion (Mao et al., 2018). According to these concepts, one inference is that molecular diffusion may occur both intracellularly and extracellularly. In addition, the compartment for diffusion is much smaller than that for perfusion, since the space in which perfusion occurs in the microcirculation is the smallest; thus, diffusion is more sensitive than perfusion to CM. Two possible explanations may account for the *D* shifts of all four groups. On the one hand, compared with CM, saline (Groups C and D) hardly affected the *D* value. On the other hand, the HGS of DM (Group C) seemed to have little effect on the *D* value when the rats were given with saline (Group D), but the effect became stronger when the rats were given CM (Groups A and B). Based on this finding, CM appears to have a greater effect on the *D* value than HGS. Greater viscosity of CM is associated with more severe renal injury (Wang et al., 2017). The high viscosity of CM enhances blood flow resistance, expands tubules, increases tubular hydrostatic pressure and interstitial vessel congestion, and decreases renal perfusion and oxygen (Wang et al., 2014). Under these circumstances, HGS is an additional activator of CI-AKI that increases the viscosity of blood and other body fluids, although it cannot provide a perfect explanation for the overall complicated pathophysiological changes. Some studies have suggested that high blood glucose concentrations increase the viscosity (Irace et al., 2014; Mushtaq et al., 2019). Physiologically, in rats, DM and HGS maintain kidney oxygen consumption at elevated levels and increase the sensitivity of kidneys to diffusion and perfusion. The responsible mechanisms include insulin resistance and activation of renin–angiotensin system (RAS), sympathetic nervous system, and oxidative stress (Zornitzki et al., 2007; Miyata et al., 2011; Li et al., 2016). Some studies have shown that the viscosity and osmolality of CM both contribute to nephrotoxicity (Liu et al., 2014). CM toxicity appears at 15 min and peaks at 3 h (Briguori et al., 2011). In addition, it seems that the viscosity of body fluids (both extracellular and intracellular fluids) has some association with glucose levels. In other research, the sensitivity of *D*^∗^ to vascular blood and different pathological changes has been found to be elevated in the course of CI-AKI. *f* is the ratio of molecular perfusion and diffusion in vessels tubules or the interstitial space. Therefore, combining *D*^∗^ and *f* may improve the accuracy of kidney disease diagnosis (Zhang et al., 2018).

A limitation of this part of study was that the rats were subjected to fMRI once and then sacrificed; thus, differences within individuals could not be assessed, undermining the reliability of the results. The results would have been more persuasive if we had performed fMRI five times in the same rat. We also did not dehydrate rats before CM injection, so the renal injury was not absolute. The lack of dehydration helped our model mimic clinical conditions. There was little difference among the three ROIs of our study, but more advanced MRI technology and imaging processes are required to improve reliability and repeatability. In conclusion, some alterations in renal function can be monitored by BOLD and IVIM imaging within 24 h after administration of CM; and *R*2*^∗^*, *D*, and *f* values have certain correlations with pathological outcomes, such as HE scores and HIF-1α expression. Compared with traditional Scr or BUN assessment, fMRI is a potential noninvasive method for earlier detection of lesions in the kidneys.

The abnormal fMRI finding may have been attributable to various pathological alterations after CM administration, especially in the DM models. The gross anatomical sections of kidney are shown in Figure 2. The CO and medulla have the clearest boundaries in DM+CM rats. The HGS might result in a darker CO (Groups A and C), while CM results in a lighter medulla (Groups A and B). The abnormal microstructure of the kidney shown in Figure 5 was strongly suggestive of CI-AKI, and this injury seemed irreversible within 72 h in Group A (Figure 6). DM first caused the kidneys to experience a hyperosmotic environment, and the renal parenchyma and interstitial tissue became loose (Figure 5, Group C). After CM was administered, the tubules and glomeruli of Groups A and C were more sensitive to any of the various mechanisms of CI-AKI than those of the groups with normal kidneys. However, CM was still the dominant cause of renal damage, as shown in Figure 6, as indicated by the lack of a dramatic difference between Groups A and B within 48 h. HIF-1α expression was also more severely affected in Group A than in Group B (Figures 7, 8). The hypoxia and related ROS generation induced by CM caused HIF-1α to accumulate in the kidneys. Enhancement of HIF-1α increases the transcription of IL-1β, which is a downstream cytokine in the Nlrp3 pathway (Tannahill et al., 2013). In our study, HIF-1α began increasing in the first 1 h after CM injection, but it decreased earlier in Group B than in Group A, which means that HGS is a positive cause of CI-AKI. HIF-1α and ROS represent damage-associated molecular patterns (DAMPs) to some extent. In addition, IL-1β and IL-18 also reached their peaks within 72 h. In comparison, Groups C and D, which were treated with saline, did not show gradual increases in Nlrp3, caspase-1, IL-1β, or IL-18 levels.

Almost all renal diseases involve expression of common cytokines and infiltration of immune cells, which indicates that the kidneys are the targets of the immune response. The role of the activated NLRP3 inflammasome in the physiopathology of CKD has been illustrated (Kurts et al., 2013). Inflammation mediated by Nlrp3 is recognized to be involved in the development of kidney injury, and urate and lipids are generally considered DAMPs (Wada and Makino, 2016). In an STZ-induced rat model of diabetic nephropathy (DN) with hyperuricemia and dyslipidemia, overexpression of NLRP3 inflammasome components [apoptosis-associated speck-like protein (ASC) and caspase-1] has been found to be associated with elevated IL-1β and IL-18 levels (Wang et al., 2012). In a genetic murine CKD model, ablation of Nlrp3 results in attenuation of tubular injury and reductions in leukocyte infiltration and renal fibrosis (Vilaysane et al., 2010). In hyperhomocysteinemia, activation of the NLRP3 inflammasome has been shown to occur in the contexts of podocyte damage and glomerular sclerosis, and reductions of NADPH oxidase levels, knockdown of ASC, or inhibition of caspase-1 may have a protective effect (Zhang et al., 2012; Abais et al., 2013, 2014). The roles of inflammasomes in DM have also been discovered, as NLRP3 activation has been observed in diabetic patients as well as in the contexts of podocytes and endothelial cell injury (Boini et al., 2014). Type 2 DM and its complications are related to mitochondrial dysfunction and oxidative stress. Mitochondria are the primary sites of ROS production, and HGS-associated impairment of mitochondria in various tissues, such as the liver, kidneys, and muscles, or in different cell types, including leukocytes, has been detected (Rovira-Llopis et al., 2018). Studies have found that HGS may participate in the occurrence of DN by regulating the activation of the NLRP3 inflammasome (Chen et al., 2016; Wada and Makino, 2016; Fu et al., 2017).

Inflammasomes are sensors of innate immunity that are triggered in response to danger signals (Schroder and Tschopp, 2010; Lamkanfi and Dixit, 2014). Triggering includes two main steps: priming and activation (Swanson et al., 2019). The NLRP3 genes are expressed in renal dendritic cells and macrophages (Martinon et al., 2009; Lichtnekert et al., 2011). High expression of HIF-1α, generation of ROS, or any other mechanisms of CI-AKI illustrated in recent decades can initiate innate immune reactions through the NLRP3 inflammasome. The NLRP3 inflammasome originates in two different ways, as demonstrated in previous studies (Lau et al., 2018; Xiang et al., 2020). Noncanonical Nlrp3 signaling functions in tubular cells and leads to apoptosis through caspase-8, while canonical Nlrp3 signaling is implicated in phagocytes and leads to pyroptosis through caspase-1/IL-18/IL-1β. Our studies (Figures 11, 12) displayed a stronger connection between Nlrp3 and caspase-1 than between Nlrp3 and caspase-8. Groups A and B were exposed to CM, and Nlrp3 expression was elevated after 48 h. The difference between these two groups was that the Nlrp3 expression in Group B was decreased at 72 h, revealing that DM contributes to innate immune activity and implying that DM is a potential predisposing factor for CI-AKI. Caspase-1, which is a downstream protein related to pyroptosis, had the same alteration, although the alteration in caspase-8 expression occurred prior to the Nlrp3 and caspase-1 alterations, indicating that it was a precursor of pyroptosis. However, a controversy exists. Some studies have indicated that caspase-8 is responsible for NLRP3 inflammasome activation in priming signals (Allam et al., 2014; Gurung et al., 2014). Chung et al. (2016) proposed that caspase-8 can form a protein complex with Nlrp3 and ASC to regulate apoptosis in a noncanonical manner in epithelial cells. In macrophages, caspase-8 facilitates pro-IL-1β production and canonical and non-canonical NLRP3 inflammasome activation (Gurung et al., 2014). Moreover, caspase-8 is able to regulate the NLRP3 inflammasome under some conditions (Kang et al., 2015; Lawlor et al., 2015). The scaffold function of caspase-8 has also been shown to be involved in the double-stranded RNA (ds-RNA)-induced activation of the NLRP3 inflammasome in macrophages (Fritsch et al., 2019). Other studies have pointed out that caspase-8 can be activated by NLRP3 inflammasomes in macrophages or dendritic cells (Sagulenko et al., 2013) or that NLRP3 inflammasomes engage caspase-8 as an important effector of innate immune signaling responses (Antonopoulos et al., 2015). It is still not clear whether caspase-8 exerts its effect on CI-AKI before Nlrp3 and caspase-1 exert their effects.

Overall, the evidence indicates that pattern recognition receptors (PRRs) in DM may respond to DAMPs formed by CI-AKI. These effects overlap with some pathophysiological changes in hyperglycemia (Li and Ren, 2020). DM and CI-AKI can both be seen as ROS generators, and ROS are important factors in the production of DAMPs. Then, DAMPs activate inflammasomes, including the NLRP3 inflammasome (Kelley et al., 2019), which leads to the expression of caspase-1, IL-1β, and IL-18. Both neutrophils and M1 macrophages are important components of the innate immune system. PRRs are commonly expressed on these immune cells. Our results showed that the numbers of neutrophils and M1 macrophages were increased after CM administration (**Figures 13-1/2**) and that DM facilitated these increases (**Figures 13-3/4**). The increases in immune cell numbers coincided with the expression of Nlrp3 and caspase-1, which reveals that pyroptosis occurs during the development of CI-AKI. The functions of phagocytes are to remove dead cells and CM or its metabolites (Lau et al., 2018).

In conclusion, fMRI BOLD and IVIM-*D* can be effective noninvasive approaches for prediction of CI-AKI; in addition, the innate immune response affects the progression of CI-AKI, and HGS exacerbates this effect. CM administration causes tubular hypoxia (as indicated by BOLD imaging and HIF-1α upregulation) and ROS generation, and ROS and dying cells can be regarded as DAMPs that are recognized by RPPs on phagocytic cells (canonical Nlrp3 pathway) and tubular cells (noncanonical Nlrp3 pathway). DM or HGS is another condition that causes ROS generation and may undermine renal function through the NLRP3 inflammasome to assists in CI-AKI development. The limitation of our study is that we did not differentiate NLRP3 inflammasomes existing in kidney-related cells from those existing phagocytes. The association between caspase-1 and caspase-8 is also unclear, but elucidating this association is far beyond this specific field of radiology. Our next research will involve *in vitro* experiments so that we can obtain deeper knowledge of the role of the NLRP3 inflammasome in CI-AKI.

## Data Availability Statement

The original contributions presented in the study are included in the article/Supplementary Material, further inquiries can be directed to the corresponding authors.

## Ethics Statement

The animal study was reviewed and approved by Xiamen University Laboratory Animal Center. Profile No. XMULAC20190005.

## Author Contributions

YL contributed to the total work of this research. HZ and SW contributed to the imaging acquiring. DS contributed to the fMRI data analyzing. XY contributed to diabetes model making. RW contributed to sample collection. YL analyzed the data and drafted the manuscript. KR guided the design of this study and revised the manuscript. All authors contributed to the article and approved the submitted version.

## Conflict of Interest

The authors declare that the research was conducted in the absence of any commercial or financial relationships that could be construed as a potential conflict of interest.

## Footnotes

1 https://github.com/MIC-DKFZ/MITK-Diffusion

## Supplementary Material

Supplementary Figure 1 Body weight changes. After high fat and sugar feeding for several weeks, the body weight of DM rats got much heavier than regular feeds group (^∗∗∗^*P* < 0.0001). DM, diabetes mellitus.

Supplementary Figure 2 Blood glucose changes. After intraperitoneal injection of STZ, blood glucose of DM model got an extreme increasing (^∗∗∗^*P* < 0.0001, ^#^*P* > 0.05). DM, diabetes mellitus.

Supplementary Figure 3 Kidney weight. Kidneys of DM rats are heavier than healthy rats (^∗∗∗^*P* < 0.0001). DM, diabetes mellitus.

Supplementary Figure 4 uMA urine microalbumin. 1, ≤10 mg/L; 2, >30 mg/L; 3, >80 mg/L; 4, ≥100 mg/L (^∗^*P* < 0.05, ^∗∗^*P* < 0.005).

Supplementary Figure 5 CD3 and Ly6G expression in kidney after injection for 48 h × 400. **(A)** CD3, which represents T cell marker; **(B)** Ly6G, which represents phagocyte marker.

Supplementary Table 1 Composition of high hat and sugar feeds.

## Abbreviations

ASC: apoptosis-associated speck-like protein
ANOVA: analysis of variance
BCA method: bicinchoninic acid method
BOLD imaging: blood oxygenation level-dependent imaging
BUN: blood urea nitrogen
CI-AKI: contrast-induced acute kidney injury
CKD: chronic kidney disease
CM: contrast medium
CMSC: Contrast Media Safety Committee
CO: cortex
DAB: diaminobenzidine
DAMPs: damage-associated molecule patterns
DM: diabetes mellitus
DN: diabetic nephropathy
ds-RNA: double-stranded RNA
ELISA: serum enzyme linked immunosorbent assay
ESUR: European Society of Urogenital Radiology
FBS: fetal bovine serum
fMRI: functional magnetic resonance imaging
GAPDH: glyceraldehyde-3-phosphate dehydrogenase
H&E: hematoxylin and eosin
HGS: high glucose status
HRP: horseradish peroxidase
HIF-1α: hypoxia-inducible factor-1 α
IL-18/IL-1β: interleukin-18/interleukin-1 β
IHC: immunohistochemistry
ISOM: inner stripe of the outer medulla
IVIM: intravoxel incoherent motion
NADPH: nicotinamide adenine dinucleotide phosphate
NLRP3: NOD-like receptor pyrin 3
OSOM: outer stripe of the outer medulla
PC-AKI: post-contrast acute kidney injury
PRRs: pattern recognition receptors
RAS: renin–angiotensin system
ROI: region of interest
ROS: reactive oxygen species
Scr: serum creatinine
SD rats: Sprague–Dawley rats
SDS: sodium dodecyl sulfate
SI: staining intensity
STZ: streptozotocin
T2WI: T2-weighted imaging.
