# Supplementary material for: The Application of Functional Magnetic Resonance Imaging in Type 2 Diabetes Rats With Contrast-Induced Acute Kidney Injury and the Associated Innate Immune Response
Source: Front Physiol. 2021 Jun 29;12:669581. doi: 10.3389/fphys.2021.669581 (PMC8276794; doi:10.3389/fphys.2021.669581)
Supplement: Supplementary Figure 1 — Body weight changes. After high fat and sugar feeding for several weeks, the body weight of DM rats got much heavier than regular feeds group (∗∗∗P < 0.0001). DM, diabetes mellitus. [file Data_Sheet_1.pdf]

Production	H10045	
	Mass ratio (g %)	Energy ratio (kcal %)
protein	24	20
carbohydrate	41	35
fat	24	45
total		100
Kcal/g	4.73	
Composition	g	kcal
Casein	233.06	932.24
Cystine	3.50	14
cornstarch	84.83	339.32
maltodextrin	116.53	466.12
sucrose	201.36	805.44
cellulose	58.26	0
Soy oil	29.13	262.17
lard	206.84	1861.56
Mineral mixture M1002	11.65	0
Calcium monophosphate	15.15	0
Calcium carbonate	6.41	0
Potassium citrate	19.23	0
Vitamin mixture V1001	11.56	46.24
Choline bitartrate	2.33	0
Food red dye	0.058	0
Total	1000	4727.09

Supplementary Table 1. Composition of high hat and sugar feeds.

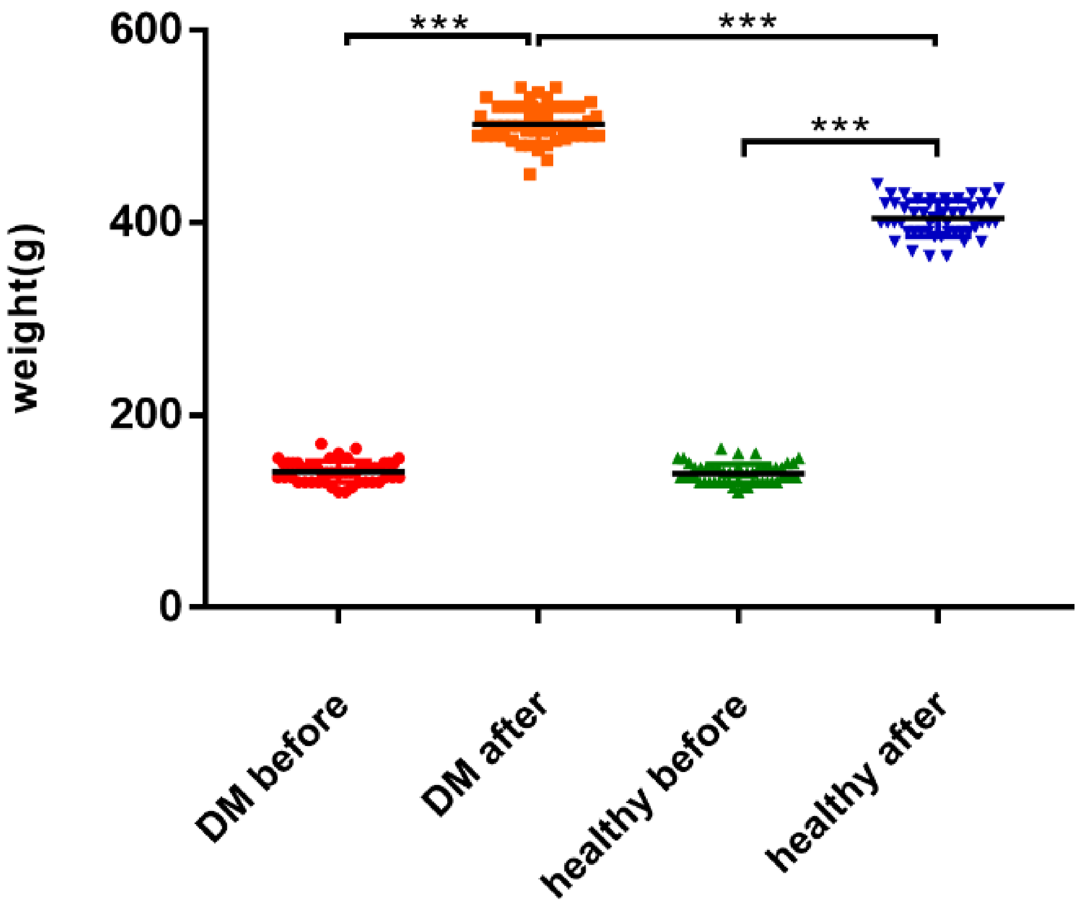

Supplementary Figure 1. Body weight changes. After high fat and sugar feeding for several weeks, the body weight of DM rats got much heavier than regular feeds group (***P<0.0001). *DM, diabetes mellitus.*

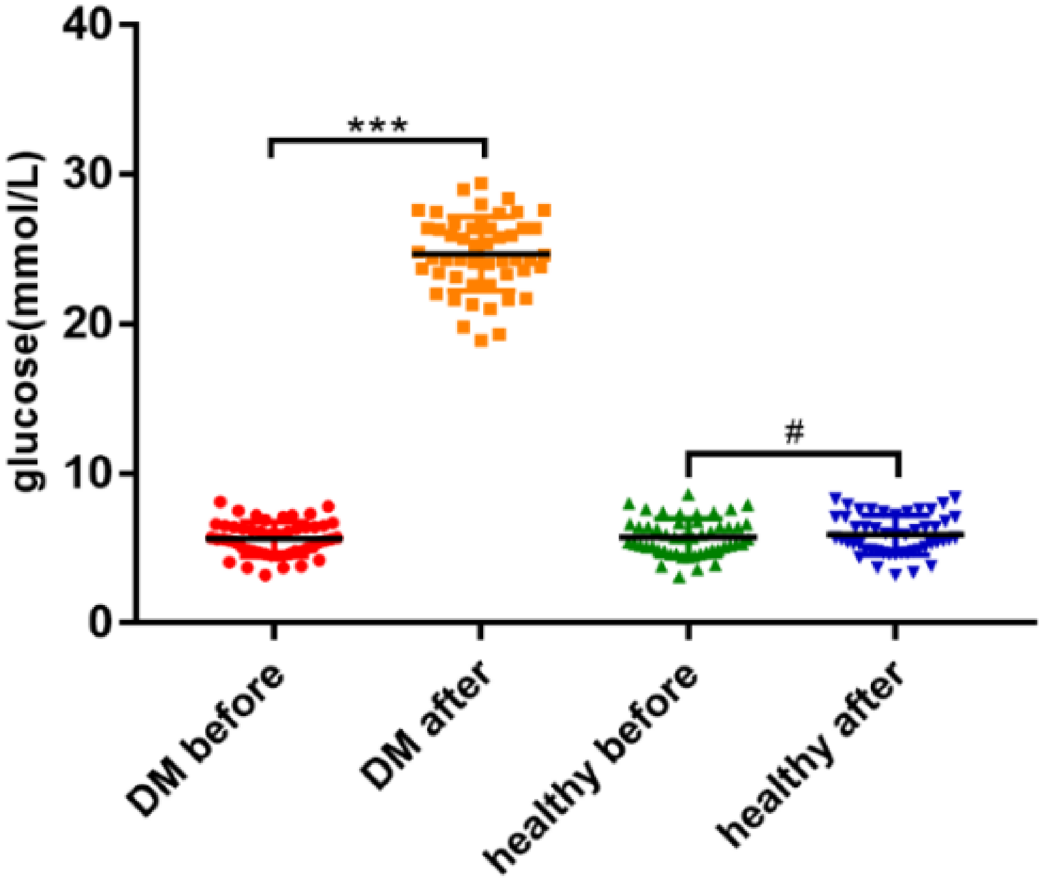

Supplementary Figure 2. Blood glucose changes. After intraperitoneal injection of STZ, blood glucose of DM model got an extreme increasing (***P<0.0001, #P>0.05). *DM, diabetes mellitus.*

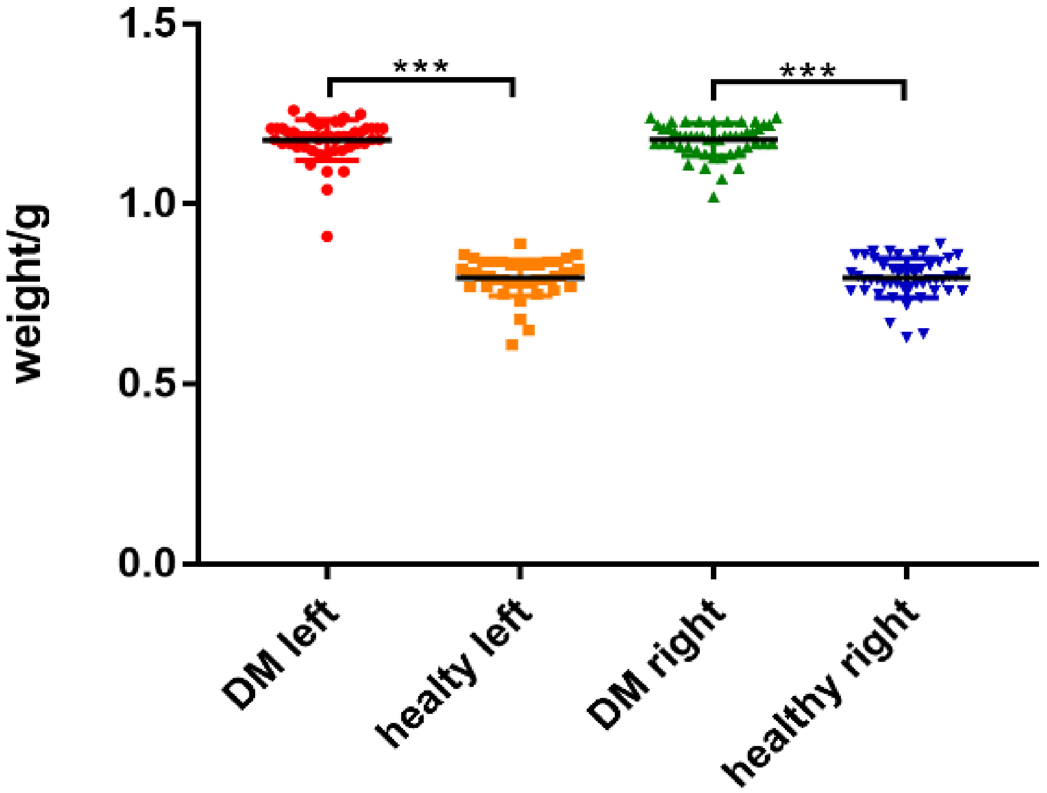

Supplementary Figure 3. Kidney weight. Kidneys of DM rats are heavier than healthy rats (***P<0.0001). *DM, diabetes mellitus.*

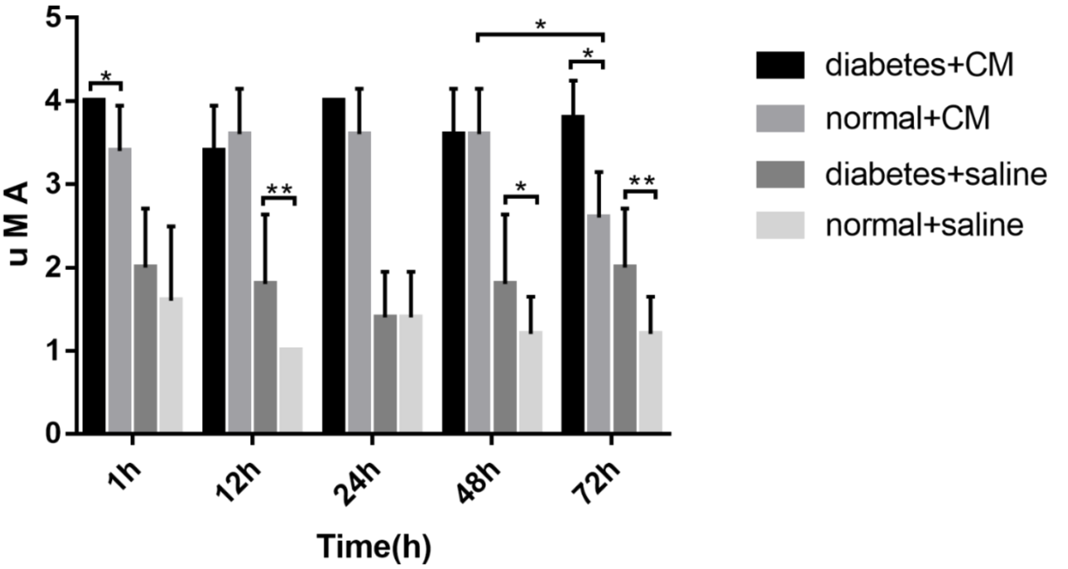

Supplementary Figure 4. uMA urine microalbumin. 1, ≤10 mg/L; 2, >30 mg/L; 3, >80 mg/L; 4≥100 mg/L. (*P <0.05, **P < 0.005).

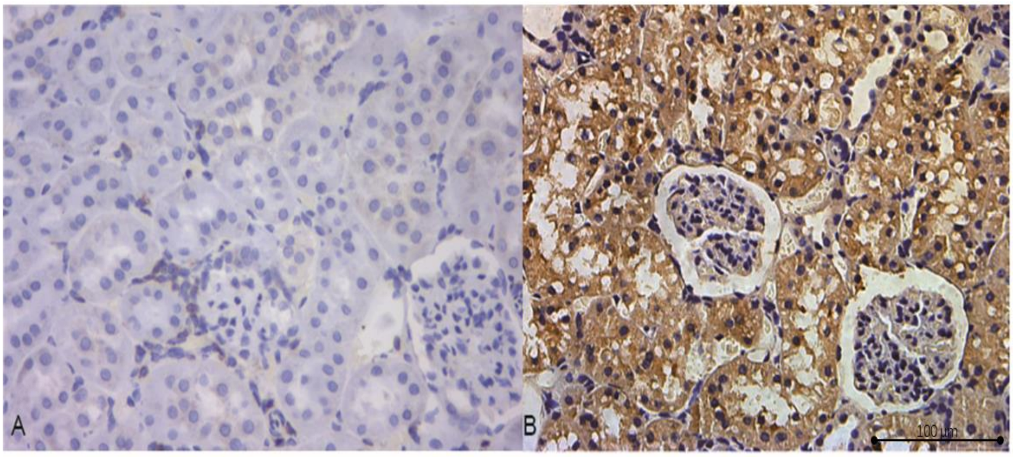

Supplementary Figure 5. CD3 and Ly6G expression in kidney after injection for 48 h *×400.*

A, CD3, which represents T cell marker； B, Ly6G, which represents phagocyte marker.
